# Novel EGFR-targeted strategy with hybrid peptide against oesophageal squamous cell carcinoma

**DOI:** 10.1038/srep22452

**Published:** 2016-03-09

**Authors:** Osamu Kikuchi, Shinya Ohashi, Tomohisa Horibe, Masayuki Kohno, Yukie Nakai, Shin’ichi Miyamoto, Tsutomu Chiba, Manabu Muto, Koji Kawakami

**Affiliations:** 1 Department of Gastroenterology and Hepatology, Kyoto University Graduate School of Medicine, Kyoto 606-8507, Japan; 2 Department of Therapeutic Oncology, Kyoto University Graduate School of Medicine, Kyoto 606-8507, Japan; 3 Department of Pharmacoepidemiology, Graduate School of Medicine and Public Health, Kyoto University, Kyoto 606-8501, Japan

## Abstract

Epidermal growth factor receptor (EGFR) is a key molecule in the pathophysiology of oesophageal squamous cell carcinoma (OSCC). However, EGFR-targeted agents such as anti-EGFR antibody or tyrosine kinase inhibitors for OSCC have not demonstrated any clinical benefits. Recently, a novel chemotherapeutic agent, EGFR(2R)-lytic hybrid peptide, a composite of EGFR-binding peptide and lytic peptide fragments, has been shown to exhibit a potent anti-tumour effect against cancers that express high EGFR levels. In this study, we investigated the validity of employing EGFR(2R)-lytic hybrid peptide against OSCC cells both *in vitro* and *in vivo*. Additionally, the toxicity of this peptide was assessed in mice. We found high EGFR expression levels on the cell surface of OSCC cells, and the EGFR-binding peptide fragment showed high affinity for OSCC cells. A potent cytotoxic effect was induced within 30 minutes by the exposure of OSCC cells to EGFR(2R)-lytic hybrid peptide. Furthermore, EGFR(2R)-lytic hybrid peptide markedly suppressed the tumour growth of OSCC cells in a xenograft model. Moreover, it did not cause any identifiable adverse effects in mice. Taken together, EGFR(2R)-lytic hybrid peptide was shown to be a valid therapeutic agent against OSCC, providing a crucial rationale regarding novel EGFR-targeted therapies against OSCC.

Oesophageal squamous cell carcinoma (OSCC) is a major histologic type of oesophageal cancer^1^, and the key therapy against advanced or metastatic OSCC is chemotherapy^2^,^3^,^4^. However, the therapeutic effect of anti-cancer agents that can be administered to treat OSCC is still limited. Indeed, combination chemotherapy with 5-fluorouracil (5-FU) and a platinum-based drug for metastatic OSCC patients has been reported to lead to response rates of 30–40%^5^,^6^,^7^. In addition, docetaxel induced a partial response in only 20% of OSCC patients^8^.

Epidermal growth factor receptor (EGFR) is frequently (71–88%) expressed in OSCC tissues^9^, and its expression is associated with a poor outcome^10^. Several clinical trials of EGFR-targeted therapies, such as anti-EGFR antibodies and EGFR tyrosine kinase inhibitors (TKI), have been conducted for patients with advanced OSCC; however, the blockade of EGFR signalling has not been shown to sufficiently improve the outcome of those patients^11^,^12^,^13^,^14^. These data suggest that another strategy is required to facilitate the development of EGFR-targeted therapies against OSCC.

Recently, an EGFR-lytic hybrid peptide was developed as a new agent of EGFR-targeted therapy^15^. This peptide consists of two unique components: an EGFR-binding peptide fragment and a lytic peptide fragment (Fig. 1A). The EGFR-binding peptide fragment shows high-level affinity for human EGFR on the cell surface, whereas the lytic peptide fragment exhibits an amphipathic helical structure due to the presence of lysines (K) and leucines (L), and penetrates cellular membranes^15^. Thus, EGFR-lytic hybrid peptide could kill EGFR-expressing cells through the combined process of specific binding to EGFR on the cell surface and subsequently disintegrating cell membranes^15^.

A modified version of EGFR-lytic hybrid peptide has since been developed to improve its binding ability to EGFR by changing the second amino acid of the EGFR-binding sequence from histidine (H) to arginine (R)^16^, and this was designated EGFR(2R)-lytic hybrid peptide (Fig. 1B). The specific binding ability of EGFR(2R)-lytic hybrid peptide to recombinant EGFR was revealed by a binding assay and biophysical analysis using surface plasmon resonance experiments^16^,^17^, and cytotoxic and anti-tumour effects of EGFR(2R)-lytic hybrid peptide were shown to be stronger than those of the original EGFR-lytic hybrid peptide^16^.

Based on these findings, we addressed the validity of EGFR(2R)-lytic hybrid peptide as a novel EGFR-targeted therapy against OSCC.

## Results

### EGFR expression and affinity of EGFR-binding peptide for surface of OSCC cells *in vitro*

**Figure 2:**
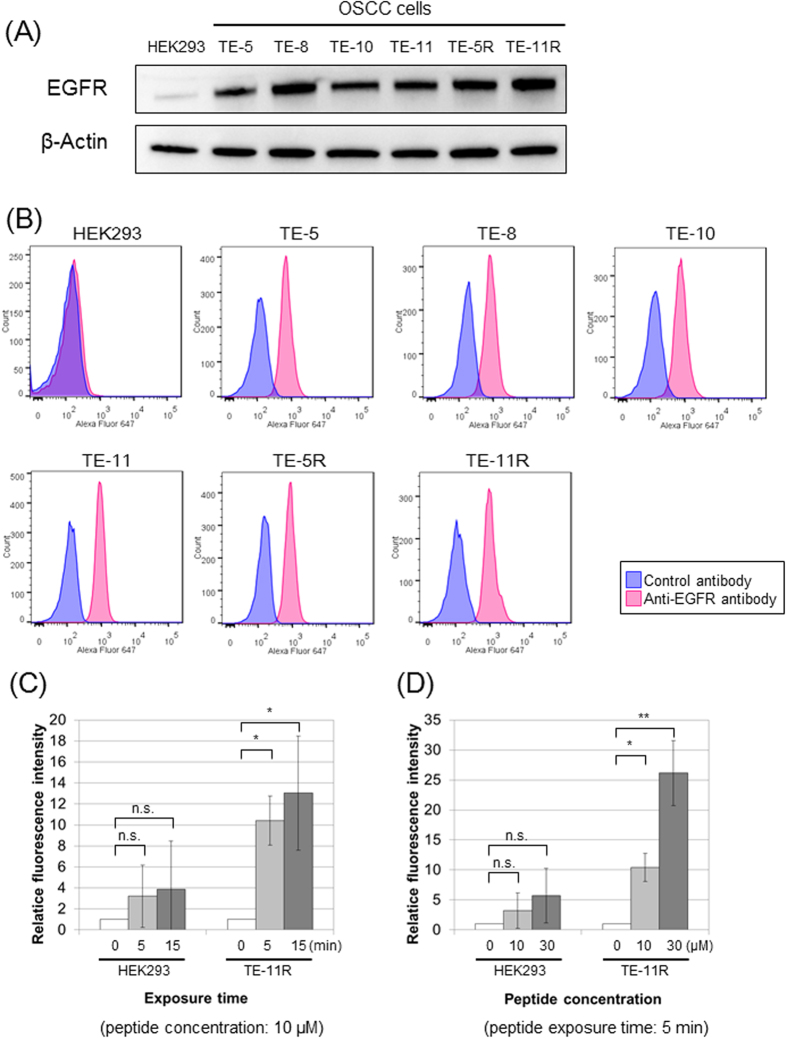
EGFR expression of OSCC cells and affinity of EGFR-binding peptide for OSCC cells. (**A**) EGFR expression levels in whole -cell lysates of HEK293 and OSCC cells (TE-5, TE-8, TE-10, TE-11, TE-5R, and TE-11R). The EGFR protein expression level was determined by Western blotting. β-Actin served as a loading control. EGFR protein was highly expressed in all OSCC cells, while its level was very low in HEK293 cells. (**B**) EGFR expressions on the cell surface of HEK293 and OSCC cells. Cells were stained with AlexaFluor 647-conjugated monoclonal anti-EGFR antibody or IgG isotype control antibody. The Fluorescence intensity determined by flow cytometry showed that EGFR was expressed on the surface of OSCC cells, whereas HEK293 cells minimally expressed it. (**C,D**) Affinity of EGFR-binding peptide for HEK293 and TE-11R cells. They were exposed to Fluorescein (FLC)-labelled EGFR-binding peptide, and the fluorescence intensity was assessed by flow cytometry. The Affinity of EGFR-binding peptide for cell membranes of TE-11R cells is increasing in time- (**C**) and dose- (**D**) dependent manners, but not in HEK293 cells. The assays were repeated three times, and data are shown as the mean ± SD. n.s.: not significant, *P < 0.05, **P < 0.01 vs. untreated (0 minutes) cells.

Firstly, we examined the expression of EGFR in whole-cell lysates of OSCC cells by Western blotting. As shown in Fig. 2A, EGFR was highly expressed in all OSCC cells, while HEK293 cells, human embryonic kidney cells, showed low-level EGFR expression. Accordingly, we used HEK293 cells as negative control cells. Next, we investigated EGFR expression on the surface of OSCC cells by flow cytometry. As with the results of EGFR expression in whole-cell lysates, EGFR was expressed on the surface of all OSCC cells, but not HEK293 cells (Fig. 2B).

Then, we examined the affinity of EGFR-binding peptide for OSCC cells (TE-11R cells) and HEK293 cells. Although EGFR-binding peptide minimally bound to HEK293 cells, the affinity of EGFR-binding peptide for TE-11R cells was significantly increased in time- (Fig. 2C) and dose- (Fig. 2D) dependent manners.

### Cytotoxicity of EGFR(2R)-lytic hybrid peptide against OSCC cells *in vitro*

**Figure 3:**
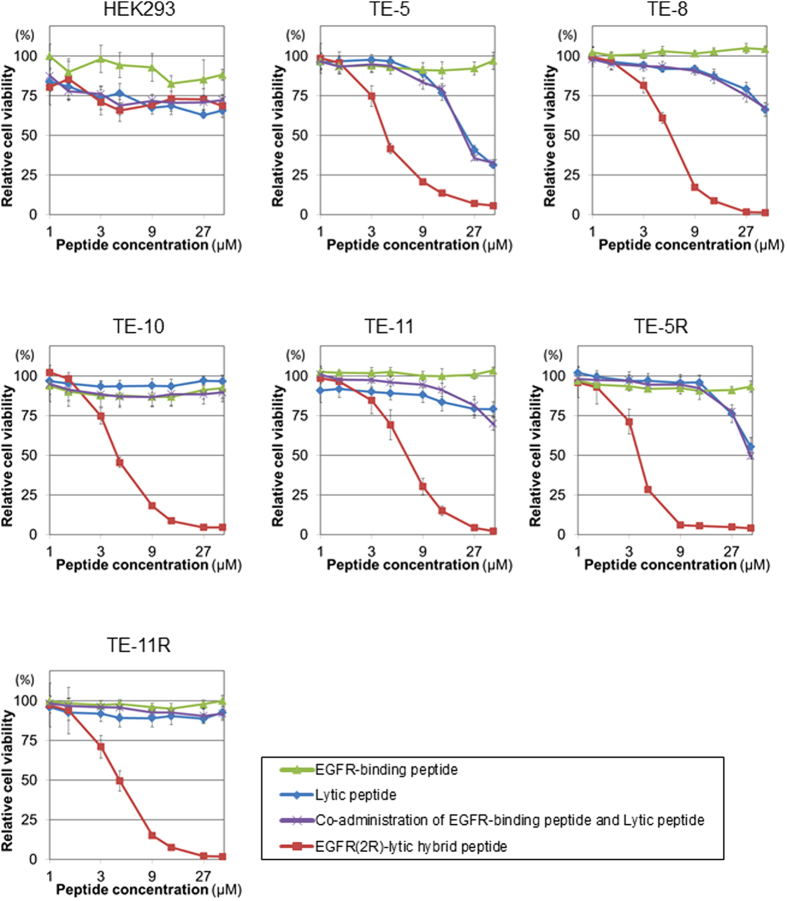
Cytotoxicity of EGFR(2R)-lytic hybrid peptide against OSCC cells *in vitro*. OSCC and control HEK293 cells were cultured with various concentrations (0–50 μM) of the EGFR-binding peptide fragment alone, lytic peptide fragment alone, co-administration of EGFR-binding peptide and lytic peptide fragments, or EGFR(2R)-lytic hybrid peptide for 30 minutes, and cell viability was measured with the WST-1 assay. A viability of 100% was defined as the amount of absorption at 450 nm found in control cells. Each point represents the mean ± S.D. (bars) from experiments conducted in sextuplicate, and the assays were repeated three times. Note that EGFR(2R)-lytic hybrid peptide exhibits a potent cytotoxicity against all OSCC cells at very low concentrations of less than 10 μM.

Cytotoxic effects of EGFR(2R)-lytic hybrid peptide, the EGFR-binding peptide fragment, lytic peptide fragment, and the co-administration of EGFR-binding peptide and lytic peptide fragments on OSCC cells were investigated. As shown in Fig. 3, EGFR(2R)-lytic hybrid peptide showed a potent cytotoxicity against all OSCC cells, whereas the cytotoxic effects of the EGFR-binding peptide fragment, lytic peptide fragment, and co-administration of EGFR-binding peptide and lytic peptide fragments were limited. The IC_50_ values of EGFR(2R)-lytic hybrid peptide for each OSCC cell are shown in Supplementary Table S1.

### Rapid disintegration of OSCC cell membranes after exposure to EGFR(2R)-lytic hybrid peptide *in vitro*

**Figure 4:**
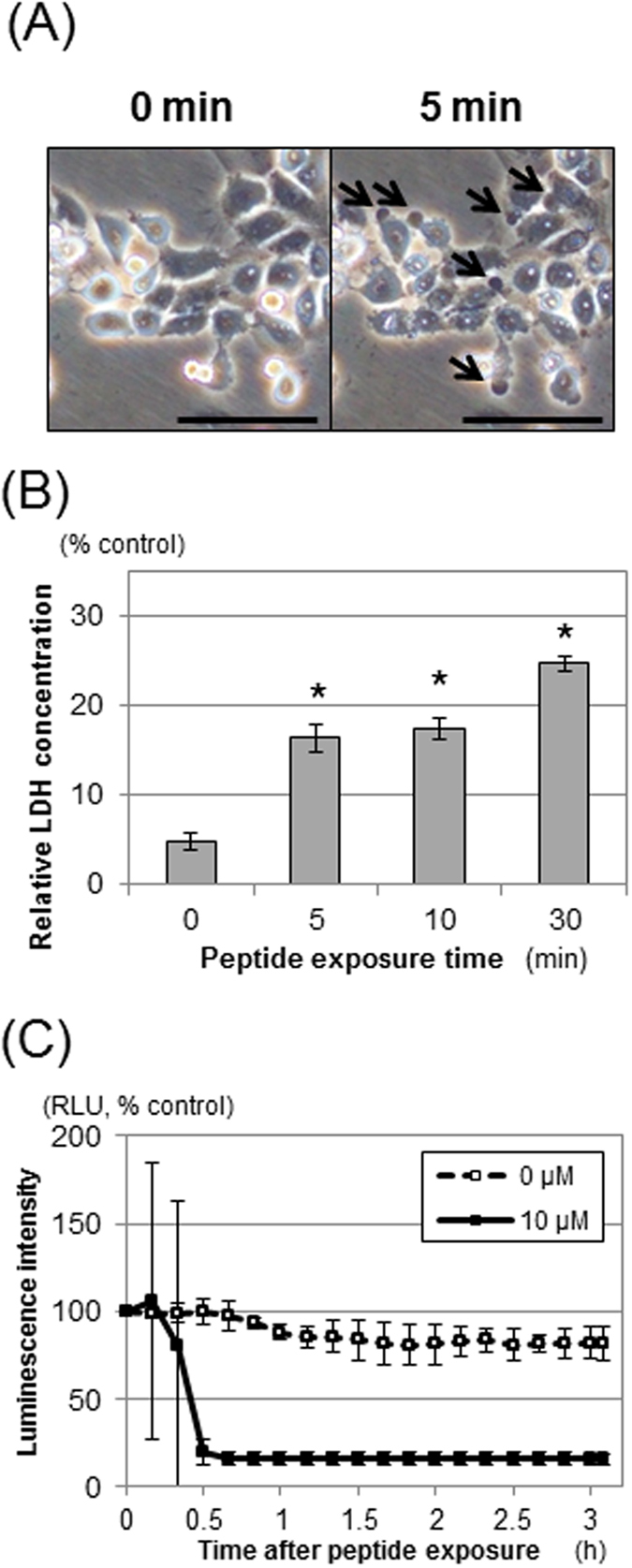
Rapid disintegration of TE-11R cell membranes after exposure to EGFR(2R)-lytic hybrid peptide *in vitro*. (**A**) Phase-contrast images of TE-11R cells after exposure to EGFR(2R)-lytic hybrid peptide (10 μM). Extravasation from cell membranes (arrows) occurred at 5 minutes after exposure to EGFR(2R)-lytic hybrid peptide. Scale bar, 100 μm. (**B**) Extracellular LDH concentrations in culture medium after exposure to EGFR(2R)-lytic hybrid peptide. The relative LDH concentration (% of positive control with Triton X-100) was calculated. A time-dependent increase of extracellular LDH levels was found after exposure to EGFR(2R)-lytic hybrid peptide (10 μM). Each point represents the mean ± S.D. (bars) from experiments conducted in sextuplicate. *P < 0.001 vs. untreated (0 minutes) cells. (**C**) The real-time cytotoxicity determined by ATP levels in TE-11R cells treated with EGFR(2R)-lytic hybrid peptide. TE-11R cells were transiently transfected with pGL4.50 vector and exposed with (right panel) or without (left panel) EGFR(2R)-lytic hybrid peptide (10 μM) for the indicated durations. Each point represents the mean ± S.D. (bars) from three cells. Note that rapid elimination of ATP was observed in the cells treated with EGFR(2R)-lytic hybrid peptide, but not in those treated without the peptide. These results indicate the rapid disintegration of the cell membrane and consequent cell death after exposure to EGFR(2R)-lytic hybrid peptide.

We examined the morphologic change of OSCC cells (TE-11R cells) after exposure to EGFR(2R)-lytic hybrid peptide. As shown in Fig. 4A, protrusion from cell membranes was observed within 5 minutes after EGFR(2R)-lytic hybrid peptide exposure. Consistently, levels of extracellular LDH, a marker of cell membrane damage, were increased in the culture medium on exposure to EGFR(2R)-lytic hybrid peptide in a time-dependent manner (Fig. 4B).

Furthermore, to assess the real-time cytotoxicity in cells treated with this peptide, we conducted the ATP bioluminescence assay. Figure 4C shows that the ATP level was reduced to near zero within 30 minutes following exposure to EGFR(2R)-lytic hybrid peptide (Fig. 4C, Supplementary Fig. S1).

### Anti-tumour effect of EGFR(2R)-lytic hybrid peptide against xenografted OSCC tumours

**Figure 5:**
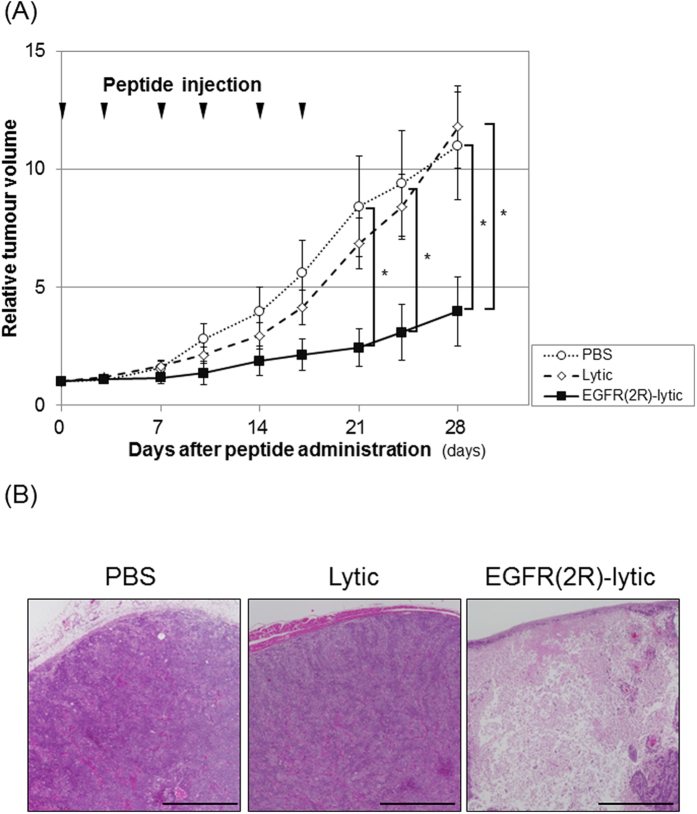
An anti-tumour effect of EGFR(2R)-lytic hybrid peptide against xenografted OSCC tumours. (**A**) Time-course volumes of xenografted tumours *in vivo*. TE-11R cells were allowed to grow for 5 weeks, and either PBS (○), lytic peptide (◇: 4 mg/kg), or EGFR(2R)-lytic hybrid peptide (▄: 4 mg/kg) was intravenously injected twice a week for a total of six doses, as indicated by the arrowheads (n = 5). The relative tumour volumes on day 28 in proportion to the volume on day 0 of the PBS, lytic peptide, and EGFR(2R)-lytic hybrid peptide groups were 11.0, 11.8 and 4.0, respectively. Each point represents the mean ± SEM (bars).*P < 0.05 vs. PBS-treated or lytic peptide-treated mice. (**B**) H&E staining of representative tumours in each group. Note that EGFR(2R)-lytic hybrid peptide induced potent tissue injury but not PBS or lytic peptide. Scale bar, 1 mm.

To investigate the anti-tumour effect of EGFR(2R)-lytic hybrid peptide against OSCC cells *in vivo*, xenografted tumours generated from TE-11R cells were treated with EGFR(2R)-lytic hybrid peptide. The EGFR(2R)-lytic hybrid peptide significantly suppressed the tumour growth of TE-11R cells (Fig. 5A) and induced tumour cell death (Fig. 5B), whereas the lytic peptide fragment without the EGFR-binding fragment did not show anti-tumour effects.

### Tolerability of EGFR(2R)-lytic hybrid peptide

**Figure 6:**
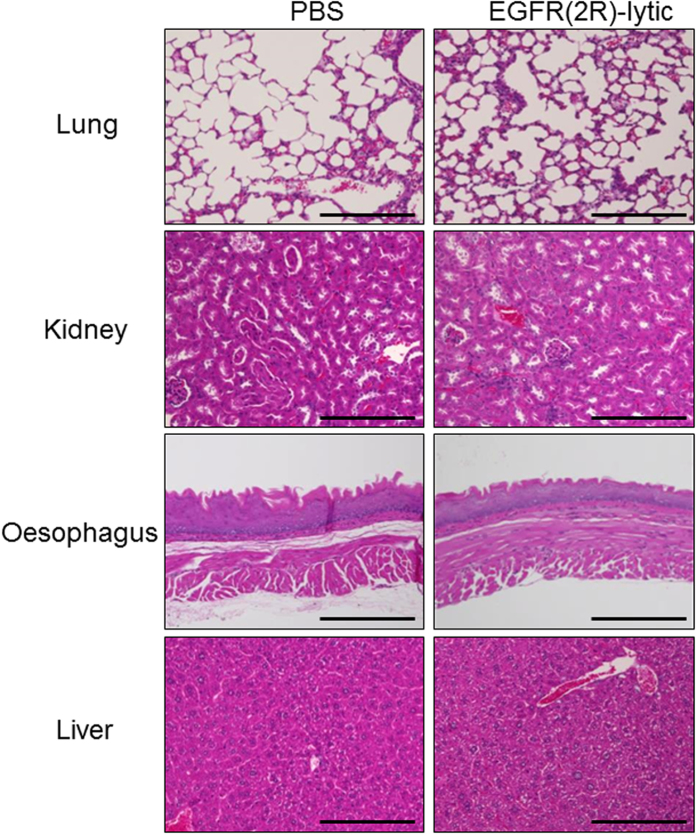
Tolerability of EGFR(2R)-lytic hybrid peptide in mice. H&E staining of the lung, kidney, oesophagus, and liver of BALB/c mice after 6-times intravenous administration of EGFR(2R)-lytic hybrid peptide (4 mg/kg). Peptides were intravenously injected twice a week for a total of six doses. Note that the administration of EGFR(2R)-lytic hybrid peptide did not affect those organs. Scale bar, 200 μm.

We assessed the toxicity of EGFR(2R)-lytic hybrid peptide in mice. Its administration led to no apparent gross abnormalities of the skin or abdominal cavities of mice. In addition, no histological damage was evident in organs (e.g., lung, kidney, oesophagus, and liver) (Fig. 6), which have been reported to express high levels of EGFR. Furthermore, neither significant haematological adverse effects nor weight loss was detected (Table 1, Supplementary Fig. S2).

**Figure 7:**
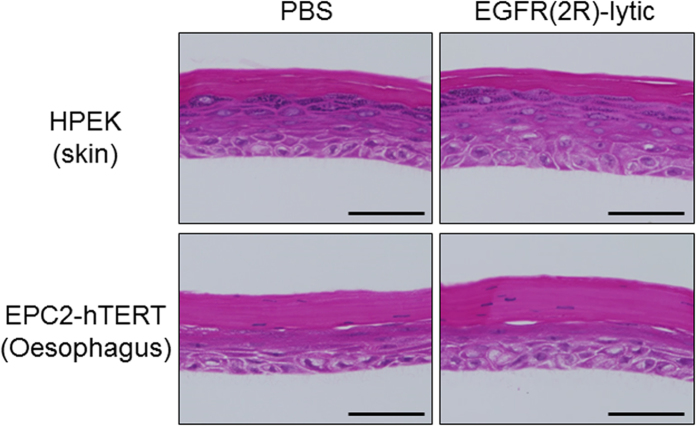
Toxic effect of EGFR(2R)-lytic hybrid peptide against stratified squamous epithelium in 3D culture. EGFR(2R)-lytic hybrid peptide (1 mg/mL) or PBS was added to the apical site of 3D-culture cell sheets consisting of HPEK (human skin primary keratinocytes) or EPC2-hTERT (immortalized human oesophageal keratinocytes) for 30 minutes. Note that exposure to EGFR(2R)-lytic hybrid peptide did not adversely affect stratified squamous epithelium derived from human skin or oesophageal keratinocytes. H&E staining. Scale bar, 50 μm.

Additionally, we tried to expose EGFR(2R)-lytic hybrid peptide on the stratified squamous epithelium. To this end, we conducted an organotypic 3D culture using human skin or oesophageal cells. Even though EGFR(2R)-lytic hybrid peptide was exposed to the stratified squamous epithelium, it did not cause any apparent histological damage (Fig. 7).

## Discussion

In the present study, we showed that all the OSCC cells we used expressed high levels of EGFR on their surface. The EGFR-binding fragment of EGFR(2R)-lytic hybrid peptide showed affinity for OSCC cells, but not HEK293 cells. EGFR(2R)-lytic hybrid peptide showed a potent cytotoxicity as well as anti-tumour effect against OSCC cells both *in vitro* and *in vivo*. Furthermore, the administration of EGFR(2R)-lytic hybrid peptide did not cause any adverse haematological or histological effects in mice. We mainly used TE-11R cells to assess the cytotoxic or anti-tumour effect of EGFR(2R)-lytic hybrid peptide, because TE-11R cells are highly transformed cells with tumourigenicity^18^.

In this study, normal oesophageal keratinocytes were not used in our *in vitro* experiments. Normal oesophageal keratinocytes are usually cultured in medium with a low calcium concentration (e.g., keratinocyte serum-free medium), in which these keratinocytes grow as undifferentiated basal cells^19^. Indeed, cultured normal oesophageal keratinocytes express high levels of cytokeratin 14 (protein expressed in the basal layers of the oesophageal stratified epithelium) but low levels of cytokeratin 13 and involucrin (proteins expressed in the suprabasal layers of the oesophageal stratified epithelium)^19^. Thus, cultured normal oesophageal keratinocytes are cells that enrich basal cells, in which EGFR is expressed^20^. Accordingly, EGFR expression in cultured oesophageal keratinocytes is high, and we could not show the difference between OSCC cells and normal oesophageal keratinocytes regarding the cytotoxicity with EGFR(2R)-lytic hybrid peptide in our *in vitro* experiments. Alternatively, the safety of EGFR(2R)-lytic hybrid peptide for the normal oesophagus was investigated by *in vivo* and organotypic 3D-culture experiments.

We showed that EGFR(2R)-lytic hybrid peptide had a higher cytotoxicity than the lytic peptide fragment. According to the report of Papo *et al.* the lytic peptide fragment forms a random coil structure in a solution, in which its ability to cause cell membrane disruption is weak^21^. However, the form of lytic peptide can be changed to an α-helical structure when it is attracted to the cell surface by static electricity due to the lipid bilayer^22^,^23^ and it exerts enhanced cytotoxicity with cell membrane disruption^21^. Notably, the EGFR expression level on the cell surface affects the cytotoxicity of EGFR-lytic hybrid peptide^15^, suggesting that the EGFR-binding peptide fragment acts as an anchor to EGFR-expressing cells, and binding of the EGFR-binding fragment with EGFR on the cell surface contributes to change the lytic peptide fragment structurally and increase membranolytic cytotoxicity. Indeed, EGFR(2R)-lytic hybrid peptide showed high-level cytotoxicity against OSCC cells, whereas it was subtle when EGFR-binding peptide and lytic peptide fragments were not hybridized (co-administration of EGFR-binding peptide and lytic peptide fragments). These results indicate that the hybridisation of EGFR-binding peptide and lytic peptide fragments plays a key role to enhance the membranolytic cytotoxicity of lytic peptide fragments.

The therapeutic effect of existing EGFR-targeting therapy on ESCC is not sufficient. In OSCC, EGFR is frequently expressed^9^, while the mutation rate is very low (1.1%)^24^. On the other hand, gene mutations and amplifications of EGFR downstream signalling pathways are frequently noted (78.6%)^24^. The therapeutic effect of existing EGFR-targeted therapies is achieved by blocking EGFR signalling in the tumour. Therefore, it is influenced by gene alteration of EGFR as well as EGFR downstream signal cascades. For example, in non-small lung cancer, response rates of EGFR-TKI are more favourable in patients with than without EGFR mutations^25^. Moreover, in colon cancer, the therapeutic effects of anti-EGFR antibody are weaker in patients with mutations of molecules downstream of EGFR than those in patients without such mutations^26^,^27^. These results suggest that the low response rate to existing EGFR-targeted therapies in OSCC patients might be due to the low frequency of EGFR mutation as well as high frequency of gene alteration of EGFR downstream signalling pathways. In this study, the anti-tumour effect of EGFR(2R)-lytic hybrid peptide is considered to depend on cell membranous EGFR expression, but not on the intracellular EGFR signalling cascades, because the pretreatment of OSCC cells with Erlotinib did not affect the cytotoxicity of EGFR(2R)-lytic hybrid peptide (Supplementary Fig. S3). Taken together, we believe that EGFR-targeted therapy using EGFR(2R)-lytic hybrid peptide is a valid strategy against OSCC.

In this study, EGFR(2R)-lytic hybrid peptide induced rapid disintegration of the cell membrane and ATP depletion in OSCC cells. Cell membrane damage with LDH leakage indicates necrotic cell death^28^, whereas ATP depletion indicates the loss of functional integrity of living cells^29^. Although our data could not determine whether cell membrane disintegration precedes or follows ATP depletion, EGFR(2R)-lytic hybrid peptide could eliminate OSCC cells effectively *in vitro*.

EGFR(2R)-lytic hybrid peptide also showed a cytotoxic effect against 5-FU-resistant TE-5R and TE-11R cells, which were established by step-wise treatment with continuously increasing concentrations of 5-FU^18^,^30^. The mechanism of 5-FU resistance in TE-5R cells is based on a gene amplification of dihydropyrimidine dehydrogenase, a 5-FU-degrading enzyme^30^. On the other hand, the mechanisms of 5-FU resistance in TE-11R cells have not been fully elucidated; however, TE-11R cells show marked tumorigenicity^18^. In this study, we showed that EGFR expression on TE-5R and TE-11R cells was comparable with that on parental TE-5 and TE-11 cells. To our knowledge, there have been no reports regarding the relationship between 5-FU resistance and EGFR expression in OSCC. We suggest that EGFR(2R)-lytic hybrid peptide could be effective against OSCC cells irrespective of 5-FU resistance as long as EGFR is overexpressed on their surface.

We suspect that EGFR(2R)-lytic hybrid peptide affects noncancerous cells that express EGFR (e.g., liver hepatocytes, respiratory epithelial cells of bronchus and lung, cells in kidney tubules, and epidermal keratinocytes of oesophagus and skin^31^,^32^); however, it did not show marked toxicity against those normal tissues in mice. Although the EGFR-binding peptide sequence was originally determined by phage display screening using human recombinant EGFR^17^, it was further modified to improve the biomolecular interaction with human EGFR^16^. The homology of the extracellular domain of human and mouse EGFR is 89% (Supplementary Fig. S4), and the EGFR-binding peptide fragment could certainly bind to mouse cells (Supplementary Fig. S5).

A possible reason why EGFR(2R)-lytic hybrid peptide has more preferential cytotoxicity against cancer cells than noncancerous cells is suggested as follows: The lytic peptide fragment has a net positive charge^21^. On the other hand, cancer cells carry more negative charges due to the presence of negatively charged phosphatidylserine than noncancerous cells^33^. Accordingly, the lytic peptide fragment itself has more affinity toward cancerous than noncancerous cells^21^. Moreover, the EGFR-binding peptide fragment is considered to contribute to bind the lytic peptide fragment with high EGFR-expressing OSCC cells and help with the α-helical structural change of the lytic peptide fragment, as we described. Thus, the selectivity of EGFR(2R)-lytic hybrid peptide is suggested to be enhanced in OSCC cells.

As a limitation, the possibility that EGFR(2R)-lytic hybrid peptide might interact with other cell surface proteins cannot be excluded. To conduct a first-in-human clinical trial, further examination regarding absorption, distribution, metabolism, and excretion as well as the metabolic stability of this peptide should be performed.

In conclusion, we conducted a preclinical study of using EGFR(2R)-lytic hybrid peptide against OSCC, and showed the validity of this peptide as a novel EGFR-targeted therapy.

## Methods

### Cell lines and cell culture

Human OSCC cell lines (TE-5, TE-8, TE-10, and TE-11) were obtained from Riken BioResource Center (Ibaragi, Japan)^34^. TE-5R and TE-11R cells, which are derived from TE-5 and TE-11 cells, respectively, are 5-FU-resistant OSCC cells that were established via exposure to parental cells with gradually increasing concentrations of 5-FU^18^,^30^. All OSCC cells were cultured in RPMI1640 medium (Life Technologies Corp., Grand Island, NY, USA), supplemented with 10% foetal bovine serum (FBS; Life Technologies.), 100 μg/mL of streptomycin, and 100 units/mL of penicillin (Life Tech.) at 37 °C in a 5% CO_2_ incubator.

HEK293, a cell line derived from human embryonic kidney cells^35^, served as low EGFR-expressing cells^36^,^37^. HEK293 was purchased from DS Pharma Biomedical Co., Ltd. (Osaka, Japan) and cultured in DMEM medium (Life Tech.), supplemented with 10% FBS, 100 μg/mL of streptomycin, and 100 units/mL of penicillin at 37 °C in a 5% CO_2_ incubator.

### Peptides

The following peptides were purchased from American Peptide Company Inc., (Sunnyvale, CA, USA) or Sigma-Aldrich Co., (St. Louis, MO, USA). Bold and italic letters are D-amino acids.

1. EGFR(2R)-lytic hybrid peptide: YRWYGYTPQNVIGGGKL***L***LK***L***L***KK***LLK***L***LKKK (American Peptide)
2. EGFR-binding peptide: YRWYGYTPQNVI (Sigma-Aldrich)
3. Lytic peptide (without EGFR-binding sequence): KL***L***LK***L***L***KK***LLK***L***LKKK (American Peptide)
4. Fluorescein (FLC)-labelled EGFR-binding peptide: FLC-YRWYGYTPQNVI (labelled with FLC at its N-terminal, Sigma-Aldrich)

### Measurement of EGFR expression

EGFR expression of the cells used in this study was measured by Western blotting and flow cytometry.

Whole-cell lysates were collected and Western blotting was performed as described previously^18^. Primary antibodies and the titres used in this study were as follows: rabbit monoclonal anti-EGFR antibody (D38B1, #4267, Cell Signaling Technology, Inc., Danvers, MA, USA; 1:1,000), rabbit monoclonal anti-β-Actin antibody (13E5, #5125, Cell Signaling; 1:5,000). β-Actin served as loading controls for whole-cell lysates.

EGFR expression on the cell surface was detected with flow cytometry by measuring the fluorointensity of dye-conjugated anti-EGFR antibody which binds to cell-surface EGFR with a minor modification from a previous report^38^. In brief, cells were washed twice with PBS, and they were stained for 30 minutes on ice with AlexaFluor 647-conjugated anti-EGFR or control antibodies in 2% FBS in PBS. Then, cells were washed twice with PBS and analysed with a flow cytometer (BD LSRFortessa Analyzer; BD Biosciences, San Jose, CA, USA). Antibodies and the titres were as follows: AlexaFluor 647-conjugated monoclonal anti-EGFR antibody (D38B1, #5588, Cell Signaling; 1:50), rabbit IgG isotype control (Alexa Fluor 647 Conjugate) antibody (#3452, Cell Signaling; 1:50). Collected data were analysed using FlowJo X software (Tree Star, Inc., Ashland, OR, USA).

### Affinity of EGFR-binding peptide to cell surface EGFR

To determine the affinity of the EGFR-binding peptide fragment to the surface of OSCC cells, the FLC-labelled EGFR-binding peptide fragment was incubated with TE-11R cells and control HEK293 cells, and the fluorointensity was measured with a flow cytometer. In brief, these cells (1 × 10^6^ cells/mL) were stained with the FLC-labelled EGFR-binding peptide fragment at the indicated times and/or concentrations on ice. After staining, these cells were washed twice with PBS, resuspended in 400 μL of ice-cold PBS, and the fluorointensity of FLC was detected through a 530/30 band-pass filter after excitation with a 488-nm blue laser.

### WST-1 assays

The cytotoxic effect of the EGFR-binding peptide fragment alone, lytic peptide fragment alone, co-administration of EGFR-binding peptide fragment with lytic peptide fragment, or EGFR(2R)-lytic hybrid peptide on OSCC cells was determined by the WST-1 assay (Roche Applied Science, Upper Bavaria, Germany) following the manufacturer’s instructions. Cells (2 × 10^4^ cells) were seeded in 96-well plates, and exposed to the indicated concentrations of peptides for 30 minutes. All data were obtained in sextuplicate with a multi-well plate reader as A_450_ – A_630_ (Infinite 200 pro, Tecan, Männedorf, Switzerland). Data are presented as the mean ± standard deviation (SD).

### LDH cytotoxicity assay

LDH is a cytosolic enzyme and is released from cells when plasma membranes are damaged^39^. Therefore, we measured the extracellular LDH concentration with the LDH Cytotoxicity Detection Kit (Takara Bio, Otsu, Japan) to detect cell membrane damage induced by EGFR(2R)-lytic hybrid peptide. In brief, OSCC cells (2 × 10^4^ cells) were plated in 96-well plates and, after 24-hour incubation, the media was changed to RPMI1640 (Life Tech.) without FBS, and they were exposed to the indicated concentrations of EGFR(2R)-lytic hybrid peptide in triplicate for 30 minutes. The media from each sample was transferred into a 96-well plate and incubated with a reaction mixture including tetrazolium salt, and the LDH concentration was measured as A_492_ – A_630_ on a multi-well plate reader.

### ATP real-time bioluminescence assay

ATP is the most important chemical energy reservoir in cells, and cell death results in a rapid ATP decrease^29^. ATP levels can be determined by assessing ATP bioluminescence with the luciferin-luciferase reaction^40^. Therefore, this assay is used to assess real-time cell viability^41^. In this study, TE-11R cells were transiently transfected with firefly luciferase-containing reporter plasmids of the cytomegalovirus promoter (pGL4.50, Promega Corp., Madison, WI, USA) using Lipofectamine LTX (Life Tech.) according to the manufacturer’s protocol, and bioluminescence images were obtained with the LUMINOVIEW LV200 luminescence imaging system (40 × objective; 5-minute intervals; 10-second exposure; Olympus Corp., Tokyo, Japan) after incubation with or without EGFR(2R)-lytic hybrid peptide. The dish was kept at 37 °C in a humidified chamber and images were taken while observing promoter activity after the addition of D-luciferin (500 μM; Promega). The regions of interest (ROIs) were selected on each cell and the luminescence intensity was measured every five minutes using LV200. Data were analysed by AQUACOSMOS ver. 2.6 software (Hamamatsu Photonics, Shizuoka, Japan).

### *In vivo* experiments

All experiments conformed to the relevant regulatory standards and were approved by the Institutional Animal Care and Use Committee of Kyoto University (Med Kyo 14523).

Xenograft transplantation was conducted as described previously^18^. Briefly, TE-11R cells (10 × 10^6^) were suspended in 50% matrigel (BD Biosciences), followed by their subcutaneous implantation into the dorsal skin of nude male mice (9 weeks of age; CLEA Japan, Inc., Tokyo, Japan). Xenografted tumours were used for the following experiments and divided into 3 groups when they had reached a tumour volume of about 50–180 mm^3^ at 40 days after injection. Tumours were free of evident necrosis at the beginning of injection. EGFR(2R)-lytic hybrid peptide (4 mg/kg), lytic peptide (4 mg/kg), or PBS was administered intravenously via the tail vein of mice twice a week for a total of six doses. The first administration day was defined as day 0. The tumours were monitored twice a week with a calliper, and the tumour volume (mm^3^) was calculated using the following formula: length x width^2^ × 0.5. The body weights were also monitored twice a week. The Relative tumour volume is shown as the Tumour volume on the indicated day/Tumour volume on day 0, and is presented as the mean ± standard error of the mean (SEM).

To assess the tolerability of EGFR(2R)-lytic hybrid peptide *in vivo*, we conducted a haematological and histological examination. We searched for anomalies in the abdominal cavity and assessed the appearance of the skin of mice, and then obtained blood and tissue samples (lung, heart, spleen, kidney, oesophagus, and liver) after 6-times administration of EGFR(2R)-lytic hybrid peptide, lytic peptide, or PBS to BALB/c mice. The body weights were monitored twice a week. Blood samples were obtained under sedation 24 hours after the final intravenous injection of EGFR(2R)-lytic hybrid peptide. Then, mice were painlessly sacrificed under appropriate anaesthesia with the inhalation of isoflurane (Escain, Mylan Pharmaceuticals, Tokyo, Japan) and cervical dislocation, and tissue samples were collected in 10% formaldehyde phosphate buffer solution (Wako Pure Chemical Industries, Ltd.) for histopathology (n = 5).

### Tolerability assessments with three-dimensional culture

To assess the safety of handling EGFR(2R)-lytic hybrid peptide in clinical situations such as preparing injection solution, we conducted an additional assessment using a three-dimensional (3D) culture model of human cells. 3D culture was established with the 3D Keratinocyte Starter Kit (3D-HPEK-50, CELLnTEC Advanced Cell Systems AG, Bern, Switzerland) following the manufacturer’s instructions. In brief, 2 × 10^5^ human primary skin keratinocytes^42^ (HPEK, CELLnTEC) and human oesophageal keratinocytes immortalized with human telomerase reverse transcriptase (EPC2-hTERT)^43^ were seeded in the insert for 3 days with CnT-PCT medium (400 μL), and then the medium was changed to 3D Prime differentiation medium inside as well as outside the insert. After overnight culture with the 3D Prime differentiation medium, 3D cultures were initiated by aspirating all the medium from inside the insert and replacing only the outside medium with fresh 3D Prime differentiation medium up to the level of the membrane, to dry the surface within the inserts. After culture with the appropriate medium for 14 days, EGFR(2R)-lytic hybrid peptide (1 mg/mL, same concentration as used in mouse experiments) or PBS was added to the apical site of 3D-culture cell sheets, and cell sheets were harvested after exposure to EGFR(2R)-lytic hybrid peptide for 30 minutes. 3D cultures were fixed with 4% paraformaldehyde phosphate buffer solution (Wako Pure Chemical Industries, Ltd.) for histopathology (n = 3).

### Statistical analyses

Data are presented as the mean ± standard deviation of the sextuplicate experiments, unless otherwise noted. Data were analysed using the 2-tailed Student’s t -test between two groups, or analysis of variance (ANOVA) between three groups. *P* < 0.05 was considered significant. All statistical analyses were performed with SPSS 19 for Windows (SPSS Inc., Chicago, IL, USA).

## Additional Information

**How to cite this article**: Kikuchi, O. *et al.* Novel EGFR-targeted strategy with hybrid peptide against oesophageal squamous cell carcinoma. *Sci. Rep.* **6**, 22452; doi: 10.1038/srep22452 (2016).

## Supplementary Material

Supplementary Information

## Figures and Tables

**Figure 1:**
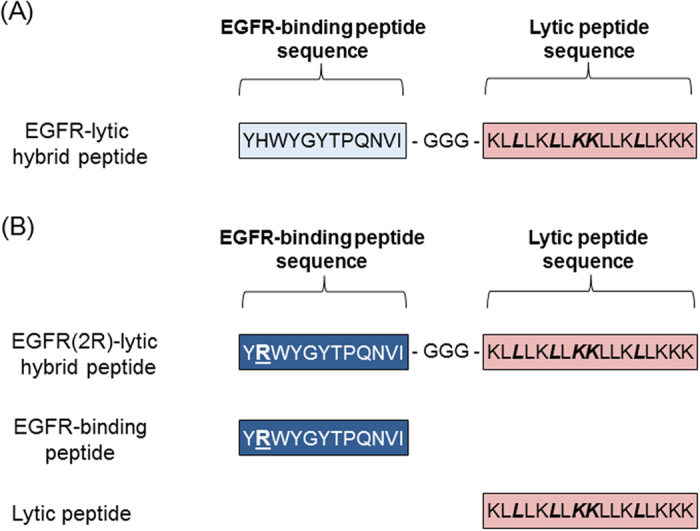
Scheme of peptides. (**A**) Original EGFR-lytic hybrid peptide consisting of an EGFR-binding peptide fragment and a lytic peptide fragment, with three glycine (G) spacers. (**B**) EGFR(2R)-lytic hybrid peptide consisting of a modified EGFR-binding peptide fragment, three glycine spacers, and a lytic peptide fragment. “(2R)” means that the second amino acid of the EGFR-binding peptide fragment is arginine (R, bold and underlined). Bold and italic letters are D-amino acids, whereas normal letters are L-amino acids. Sequences of EGFR-binding peptide and lytic peptide fragments are also shown.

**Table 1:** Haematological assessment after the administration of EGFR(2R)-lytic hybrid peptide to mice.

	EGFR(2R)-lytic	Lytic	PBS	P
WBC (×10^3^/μL)	2.5 ± 0.7	2.5 ± 0.2	3.1 ± 1.0	0.341
RBC (×10^4^/μL)	1002.2 ± 62.1	909.4 ± 138.4	907.8 ± 216.3	0.550
Hb (g/dL)	16.1 ± 0.1	15.8 ± 0.7	15.3 ± 2.1	0.593
Hct (%)	52.9 ± 3.3	49.1 ± 7.4	47.6 ± 10.5	0.549
Plt (×10^4^/μL)	81.5 ± 4.6	77.0 ± 11.6	69.8 ± 26.0	0.546

EGFR(2R)-lytic hybrid peptide (4 mg/kg) was intravenously injected into BALB/c mice twice a week for a total of six doses. Blood samples were obtained one day after the final intravenous injection of EGFR(2R)-lytic hybrid peptide. P-values were assessed by analysis of variance (ANOVA). Data are presented as the mean ± S.D. (n = 5). Note that EGFR(2R)-lytic hybrid peptide administration did not cause any haematological abnormalities in mice.
