# Supplementary material for: Novel EGFR-targeted strategy with hybrid peptide against oesophageal squamous cell carcinoma
Source: Sci Rep. 2016 Mar 9;6:22452. doi: 10.1038/srep22452 (PMC4796678; doi:10.1038/srep22452)
Supplement: Supplementary Information [file srep22452-s1.pdf]

Novel EGFR-targeted strategy with hybrid peptide against oesophageal squamous cell carcinoma

Osamu Kikuchi[1], Shinya Ohashi[2], Tomohisa Horibe[3], Masayuki Kohno[3], Yukie Nakai[2], Shin'ichi Miyamoto[1], Tsutomu Chiba[1], Manabu Muto[2], and Koji Kawakami[3]*

1: Department of Gastroenterology and Hepatology, Kyoto University Graduate School of Medicine, Kyoto 606-8507, Japan

2: Department of Therapeutic Oncology, Kyoto University Graduate School of Medicine, Kyoto 606-8507, Japan

3: Department of Pharmacoepidemiology, Graduate School of Medicine and Public Health, Kyoto University, Kyoto 606-8507, Japan

Supplementary Information

Supplementary figure legends

Supplementary Fig. S1 Real-time cell viability of single cells after exposure to EGFR(2R)-lytic hybrid peptide *in vitro*.

TE-11R cells were transiently transfected with pGL4.50 vector and time-course analysis of ATP bioluminescence was performed with (right panel) or without (left panel) exposure to EGFR(2R)-lytic hybrid peptide (10 μM) for the indicated durations. All data represent the relative luminescent intensity of the ROIs. Squares in the luminescence images indicate the ROIs. Note that luminescence had disappeared within 30 minutes following exposure to EGFR(2R)-lytic hybrid peptide. All scale bars are 200 μm.

Supplementary Fig. S2 Body weight change of BALB/c mice after exposure to EGFR(2R)-lytic hybrid peptide.

EGFR(2R)-lytic hybrid peptide (■: 4 mg/kg), lytic peptide (◇: 4 mg/kg), or PBS (○) was intravenously injected twice a week for a total of six doses, as indicated by the arrows. The body weights were monitored twice a week. Each

point represents the mean ± SEM (bars). n = 5.

Supplementary Fig. S3 Effects of inhibition of EGFR signalling on cytotoxicity of EGFR(2R)-lytic hybrid peptide

(A) Phospho-EGFR and total-EGFR expression levels in whole-cell lysates of TE-11R cells in the presence or absence of preincubation with EGFR-tyrosine kinase inhibitor (Erlotinib). The phospho-EGFR and total-EGFR protein expression levels were determined by Western blotting. The relative density of phospho-EGFR was calculated by densitometry. β-Actin served as a loading control. Phospho-EGFR protein levels in TE-11R were reduced by Erlotinib (1 μM, 60 min) preincubation.

(B) Affinity of EGFR-binding peptide for TE-8 and TE-11R cells with or without preincubation with EGFR-tyrosine kinase inhibitor. TE-8 and TE-11R cells were exposed to Fluorescein (FLC)-labelled EGFR-binding peptide in the presence or absence of Erlotinib preincubation (1 μM, 60 min). The fluorescence intensity was assessed by flow cytometry. Note that the affinity of EGFR-binding peptide to the cell membrane of those cells was not influenced by treatment with Erlotinib. The assays were repeated three times, and data are shown as the

mean ± SD.

(C) TE-8 and TE-11R cells were cultured with various concentrations (0–50 μM) of EGFR(2R)-lytic hybrid peptide for 30 minutes with or without preincubation with Erlotinib (1 μM, 60 min), and cell viability was determined by the WST-1 assay. A viability of 100% was defined as the amount of absorption at 450 nm found in control cells. Each point represents the mean ± S.D. (bars) from experiments conducted in sextuplicate, and the assays were repeated three times. Note that the inhibition of EGFR signalling does not affect the cytotoxicity of EGFR(2R)-lytic hybrid peptide against OSCC cells.

Supplementary Fig. S4 The amino acid sequence alignment of human and mouse EGFRs.

The alignment of the EGFR extracellular domain (25 - 633 amino acids) against human (NP_005219.2) and mouse (NP_997538.1) EGFRs. Amino acid sequence alignment was performed using clustalw (http://www.genome.jp/tools/clustalw/), and the identity of these sequences was confirmed as 89% consistent by NCBI Blast (http://blast.ncbi.nlm.nih.gov/Blast.cgi), respectively. " * " indicates positions that

have a single, fully conserved residue. " : " indicates that one of the following 'strong' groups is fully conserved: STA, NEQK, NHQK, NDEQ, QHRK, MILV, MILF, HY, FYW (single amino acid code). " . " indicates that one of the following 'weak' groups is fully conserved: CSA, ATV, SAG, STNK, STPA, SGND, SNDEQK, NDEQHK, NEQHRK, FVLIM, HFY (single amino acid code).

Supplementary Fig. S5 Affinity of EGFR-binding peptide for mouse-derived cells *in vitro*.

HEK293 or Colon26 cells (mouse-derived cells) were exposed to fluorescein (FLC)-conjugated EGFR-binding peptide (10 μM). The affinity of EGFR-binding peptide for cell membranes was assessed by flow cytometry. Note that EGFR-binding peptide showed an affinity for Colon26 cells.

Supplementary table legend

Supplementary Table S1. Inhibitory concentration (IC_{50}) of EGFR(2R)-lytic hybrid peptide for OSCC and HEK293 cells.

The inhibitory concentration (IC_{50}) of EGFR(2R)-lytic hybrid peptide for OSCC cells was calculated from the results of the WST-1 assay (Fig.3). Data are

shown as the mean ± S.D. of three independent experiments, each performed in sextuplicate. Note that EGFR(2R)-lytic hybrid peptide exhibits potent cytotoxicity against all OSCC cells at very low concentrations of less than 10 μM.

Supplementary methods

Primary antibodies and the titres used in the studies of Supplementary Fig. S3 were as follows: rabbit monoclonal phospho-EGFR (Tyr1068) antibody (D7A5, #3777, Cell Signaling; 1:1,000), rabbit monoclonal EGFR antibody (D38B1, #4267, Cell Signaling; 1:1,000), rabbit monoclonal anti-β-actin antibody (13E5, #5125, Cell Signaling; 1:5,000). β-Actin served as a loading control for whole-cell lysates.

Supplement Figure S1.

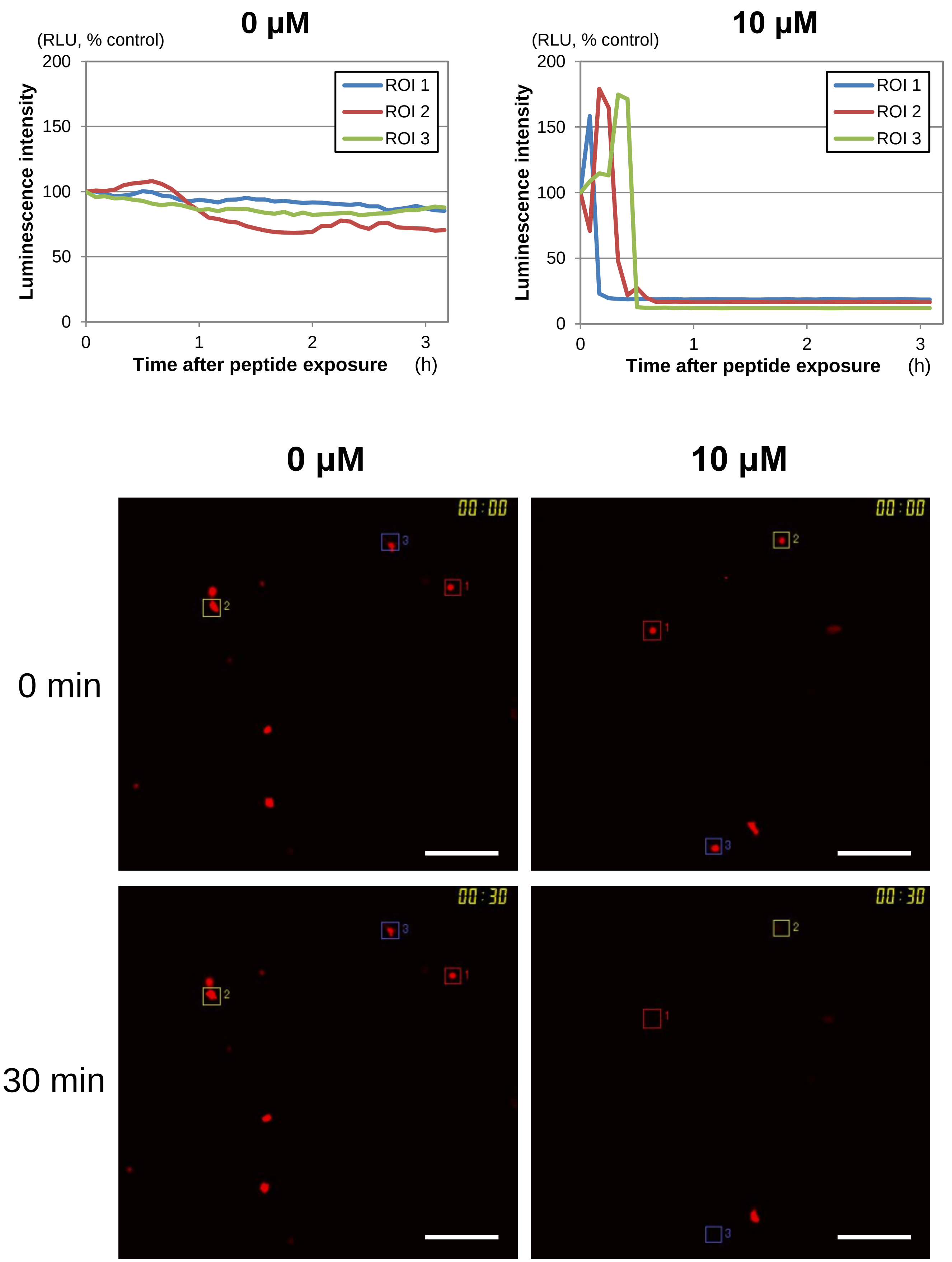

Supplement Figure S2.

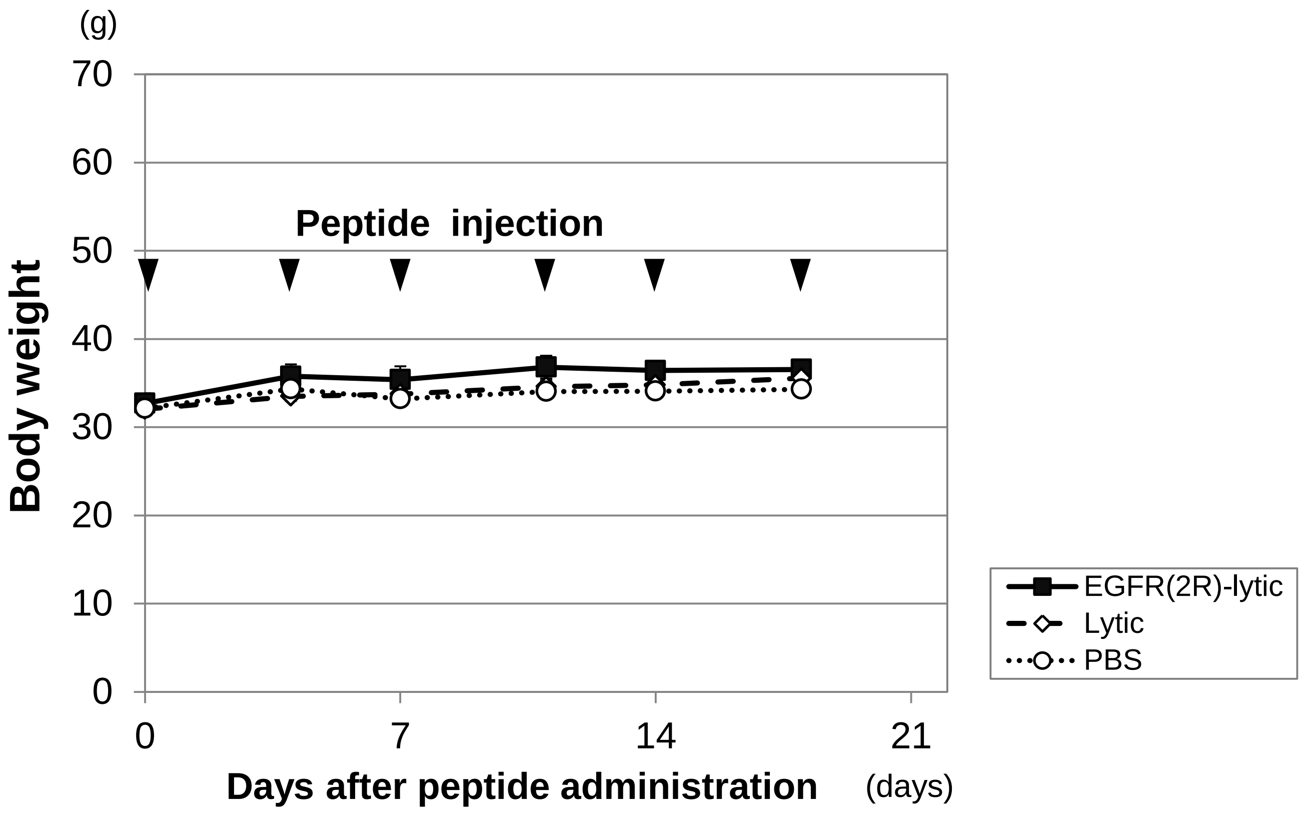

Supplement Figure S3.

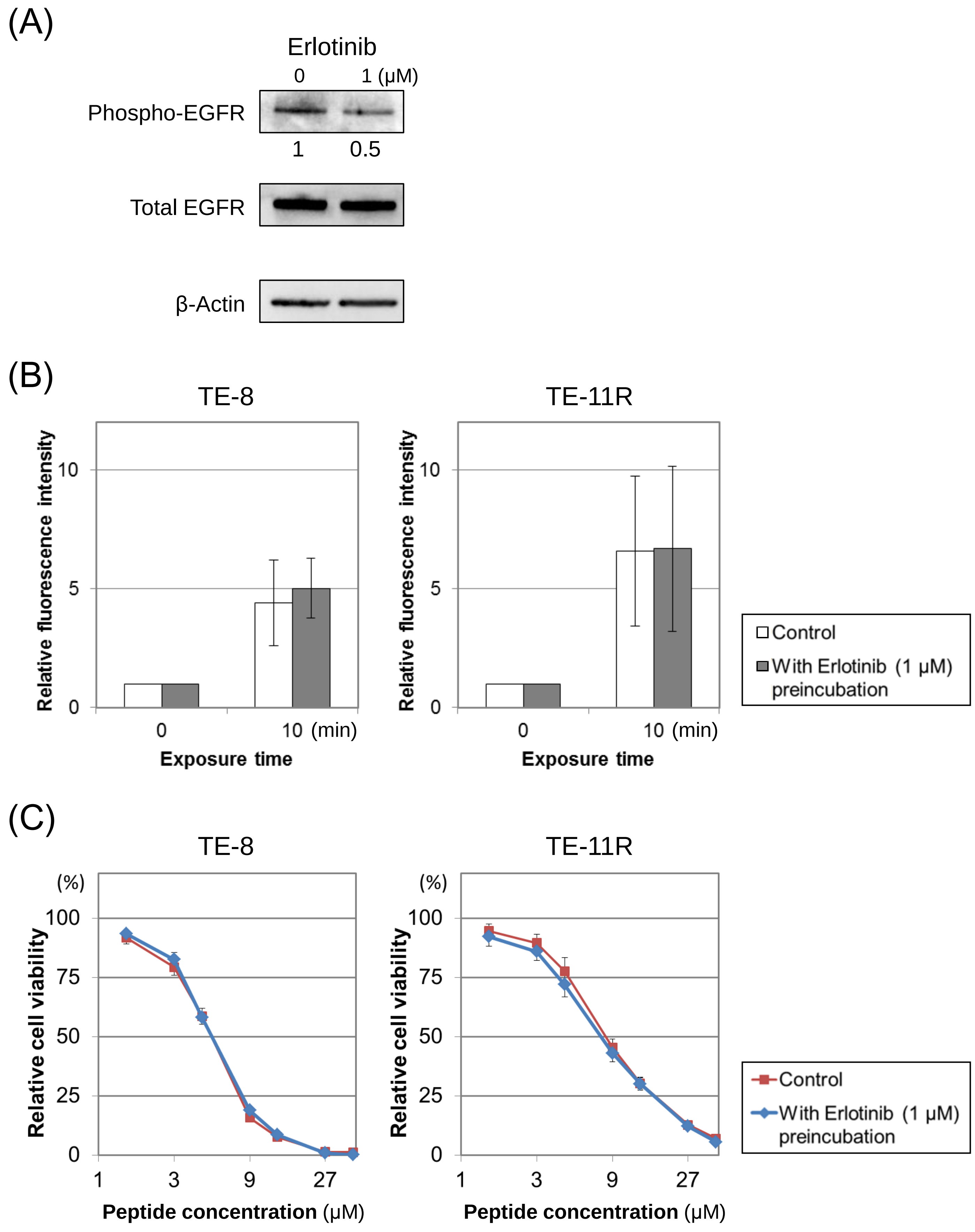

Supplement
Figure S4.

```
Human           LEEKKVCQGTSNKLTQLGTFEDHFLSLQRMFNNCEVVLGNLEITYVQRNYDLSFLKTIQE
Mouse           LEEKKVCQGTSNRLTQLGTFEDHFLSLQRMYNNCEVVLGNLEITYVQRNYDLSFLKTIQE
                ************:*****************:*****************************

Human           VAGYVLIALNTVERIPLENLQIIRGNMYYENSYALAVLSNYDANKTGLKELPMRNLQEIL
Mouse           VAGYVLIALNTVERIPLENLQIIRGNALYENTYALAILSNYGTNRTGLRELPMRNLQEIL
                **************************  ***:****:****.:*:***:***********

Human           HGAVRFSNNPALCNVESIQWRDIVSSDFLSNMSMDFQNHLGSCQKCDPSCPNGSCWGAGE
Mouse           IGAVRFSNNPILCNMDTIQWRDIVQNVFMSNMSMDLQSHPSSCPKCDPSCPNGSCWGGGE
                 ********* ***:::*******.. *:******:*.* .** *************.**

Human           ENCQKLTKIICAQQCSGRCRGKSPSDCCHNQCAAGCTGPRESDCLVCRKFRDEATCKDTC
Mouse           ENCQKLTKIICAQQCSHRCRGRSPSDCCHNQCAAGCTGPRESDCLVCQKFQDEATCKDTC
                **************** ****:*************************:**:*********

Human           PPLMLYNPTTYQMDVNPEGKYSFGATCVKKCPRNYVVTDHGSCVRACGADSYEMEEDGVR
Mouse           PPLMLYNPTTYQMDVNPEGKYSFGATCVKKCPRNYVVTDHGSCVRACGPDYYEVEEDGIR
                ************************************************.* **:****:*

Human           KCKKCEGPCRKVCNGIGIGEFKDSLSINATNIKHFKNCTSISGDLHILPVAFRGDSFTHT
Mouse           KCKKCDGPCRKVCNGIGIGEFKDTLSINATNIKHFKYCTAISGDLHILPVAFKGDSFTRT
                *****:*****************:************ **:************:*****:*

Human           PPLDPQELDILKTVKEITGFLLIQAWPENRTDLHAFENLEIIRGRTKQHGQFSLAVVSLN
Mouse           PPLDPRELEILKTVKEITGFLLIQAWPDNWTDLHAFENLEIIRGRTKQHGQFSLAVVGLN
                *****:**:******************:* ***************************.**

Human           ITSLGLRSLKEISDGDVIISGNKNLCYANTINWKKLFGTSGQKTKIISNRGENSCKATGQ
Mouse           ITSLGLRSLKEISDGDVIISGNRNLCYANTINWKKLFGTPNQKTKIMNNRAEKDCKAVNH
                **********************:****************..*****:.**.*:.***..:

Human           VCHALCSPEGCWGPEPRDCVSCRNVSRGRECVDKCNLLEGEPREFVENSECIQCHPECLP
Mouse           VCNPLCSSEGCWGPEPRDCVSCQNVSRGRECVEKCNILEGEPREFVENSECIQCHPECLP
                **:.***.**************:*********:***:***********************

Human           QAMNITCTGRGPDNCIQCAHYIDGPHCVKTCPAGVMGENNTLVWKYADAGHVCHLCHPNC
Mouse           QAMNITCTGRGPDNCIQCAHYIDGPHCVKTCPAGIMGENNTLVWKYADANNVCHLCHANC
                **********************************:**************.:******.**

Human           TYGCTGPGL
Mouse           TYGCAGPGL
                ****:****
```

Supplement Figure S5.

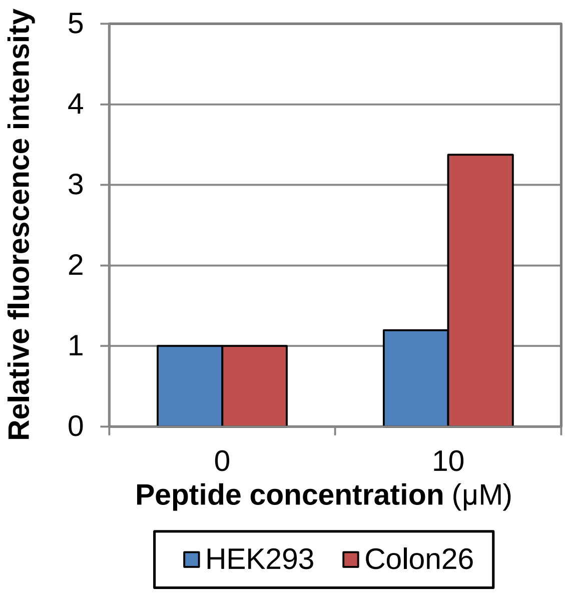

Supplement Table S1.

Inhibitory concentration (IC_{50}) of EGFR(2R)-lytic hybrid peptide

**Cell**	**IC_{50} (μM)**
HEK293	29.8 ± 8.6
TE-5	4.0 ± 0.1
TE-8	4.5 ± 0.2
TE-10	6.3 ± 0.6
TE-11	6.9 ± 0.3
TE-5R	3.6 ± 0.1
TE-11R	4.2 ± 0.4
